# CAR T-cell therapy and the onco-nephrologist

**DOI:** 10.3389/fneph.2024.1378250

**Published:** 2024-04-19

**Authors:** Marco Aurelio Salvino, Alberto Mussetti, Marta Peña, Annalisa Paviglianiti, Abel Santos Carreira, Daniel Rizky, Anna Sureda

**Affiliations:** ^1^ Programa Pos Graduacao Medicina Saude (PPGMS), Universidade Federal da Bahia, Salvador, Brazil; ^2^ L’Hospitalet, Institut Català de Oncologia, Barcelona, Spain; ^3^ Hematology Department, Instituto D´or de Pesquisa e Ensino-Bahia (IDOR Ba), Salvador, Brazil; ^4^ Hematology Medical Oncology, Dr. Kariadi General Hospital, Semarang, Indonesia; ^5^ Institut d’Investigació Biomédica de Bellvitge (IDIBELL), Universitat de Barcelona, Barcelona, Spain

**Keywords:** CAR T-cell, immune effector cells, kidney, onco-nephrology, acute kidney injury

## Abstract

Cell therapy, specifically the revolutionary chimeric antigen receptor (CAR) T-cell therapy, has transformed the landscape of oncology, making substantial strides in practical treatment approaches. Today, established guidelines for diseases such as lymphomas, myelomas, and leukemias actively advocate the utilization of these once-unconventional therapies. The practical impact of these therapies is underscored by their unparalleled efficacy, reshaping the way we approach and implement treatments in the realm of oncology. However, CAR T-cell therapy, with its performance in anti-tumor aggression through cellular action and inflammatory response, also comes with various adverse events, one of which is kidney injury. Therefore, the management of these side effects is extremely important. The integration of knowledge between oncologists and specialized nephrologists has led to the emergence of a new sub-area of expertise for onco-nephrologists specializing in managing kidney complications from immune effector therapies.

## 1 Introduction

Since 2017, the Food and Drug Administration (FDA) has granted approval for six CAR T-cell therapies designed for hematological cancers. Kymriah^®^ (tisagenlecleucel) and Yescarta^®^ (axicabtagene ciloleucel) marked the initial approvals, targeting patients with refractory/relapsed B-cell precursor acute lymphoblastic leukemia (ALL) and relapsed or refractory diffuse large B-cell lymphoma (DLBCL) after two or more lines of systemic therapies. Subsequent approvals expanded the list, with two additional agents of anti-cluster of differentiation (CD)19 CARs, namely, Tecartus^®^ (brexucabtagene autoleucal) and Breyanzi^®^ (lisocabtagene maraleucel), making four out of six CAR T-cell products with additional indications such as follicular lymphoma, high-grade B-cell lymphoma, primary mediastinal B-cell lymphoma, and mantle cell lymphoma. The CAR T-cell therapies, namely, Abecma^®^ (idecabtagene vicleucel) and Carvykti^®^ (ciltacabtagene autoleucel), focus on B-cell maturation antigen (BCMA) and target patients with relapsed or refractory multiple myeloma after four or more prior lines of therapy, and they ultimately expand the scope of CAR T-cell therapy indications across various hematological cancers (1–3).

**Figure 1:**
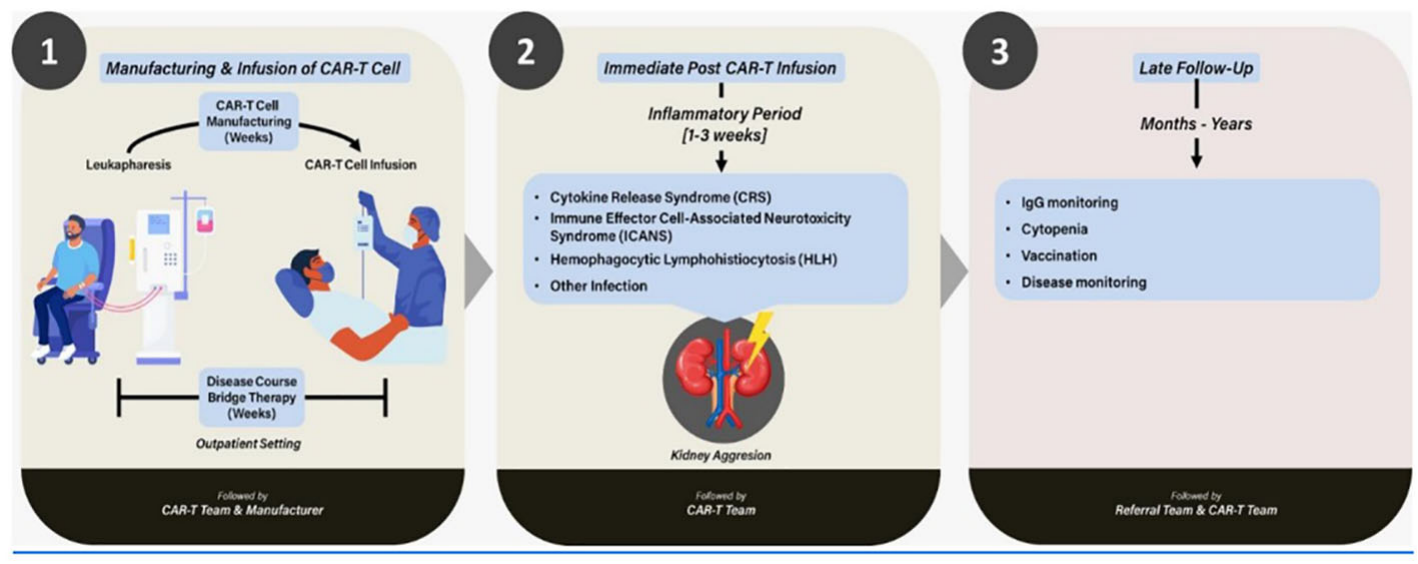
Integration of CAR T-cell therapy in the context of kidney injury: an overview.

Autologous CAR T-cell therapy is a type of cancer treatment that involves reprogramming the body’s immune cells to recognize and attack cancer cells. The treatment involves extracting a patient’s T cells, genetically modifying them to produce chimeric antigen receptors (CARs) that can recognize and bind to specific proteins on cancer cells, and then infusing the modified cells back into the patient’s bloodstream. The process of cell production, spanning patient apheresis to vein infusion, and subsequent clinical management is inherently intricate, necessitating stringent quality control measures across all facets (see **Figure 1**). The cells meticulously prepared for orchestrating an immune attack against a specific target are referred to as immune effector cells (IEC). IEC therapy has its own chapter dedicated to highly specific and specialized preparation and management in the Fact-Jacie Quality Management International Guidelines (4).

Following the infusion of CAR T cells into the patient’s bloodstream, there is a notable exponential expansion of modified T cells, concomitant with the initiation of the attack on the tumor. Insights gained from several years of clinical practice have consequently revealed the following:

- The severity of adverse events correlates positively with the aggressiveness of the disease and the extent of the tumor burden.
- Patient fragility directly correlates with heightened risks and complications associated with CAR T-cell therapy.

Each CAR T cell exhibits a distinct toxicity/efficacy profile; for instance, the CD28 costimulator domain is associated with increased acute toxicities but also higher efficacy. During the 2–3 weeks post-CAR T-cell infusion treatment period, the patient undergoes a phase of inflammation and tumor destruction. Tumor lysis, varying degrees of infection, and cytokine release syndrome collectively elevate the risks of renal dysfunction. Furthermore, in certain diseases like multiple myeloma, the kidney is frequently impacted by the underlying condition, rendering it more susceptible to functional impairments (5–11).

## 2 Article type

This is a review article discussing the interaction of the kidney with new cellular therapies and the role of the onco-nephrologist in this emerging context.

## 3 Review

### 3.1 Renal adverse events in the main CAR T-cell studies

We list the main studies related to CAR T-cell therapy, and we note that serious adverse events (SAE) (grades 3 and 4) in the studies are very low and are not a major concern. However, it is up to the nephrologist to assess which patients are most at risk (e.g., risk of tumor lysis) and to follow the usual preventive measures (e.g., adequate hydration, attention to nephrotoxic drugs, and patient needs assessment) (12–14).

The studies showed that serious adverse events (grades 3 and 4) below 5% were observed (**Table 1**), even in studies on multiple myeloma. However, an important point to remember is that in most studies, a clearance of more than 30 or 40 mL/min/1.73 m^2^ is part of the eligibility criteria. In other words, there is no relevant clinical experience for patients with previous severe renal dysfunction or patients on dialysis. In a comprehensive analysis of various CAR T-cell therapy trials, kidney adverse events were assessed across multiple indications. The KARMMA 3 trial (15), focusing on multiple myeloma, reported a 3% incidence of kidney adverse events compared to 2% on standard of care (SOC). KARMMA 1 (10) and CARTITUDE 1 (11) trials in multiple myeloma revealed no relevant findings or serious adverse events (SAE) related to the kidneys. In the JULIET trial (16) for non-Hodgkin lymphoma, an increase in blood creatinine levels was observed in 11% of the cases, with a 4% incidence of SAE. ZUMA 1 (17) trials showed no significant kidney-related findings, while in the ZUMA 7 trial (18) for non-Hodgkin lymphoma, acute kidney injury was noted in 2% of cases, with an additional 1% experiencing SAE, compared to the SOC where the incidence was 5% for all cases and 2% for SAE. The BELINDA trial (19) reported a 17.9% increase in creatinine levels, with a 1.2% incidence of SAE, in contrast to the SOC with 18.1% and 0.6% for all and SAE, respectively. Overall, the data underscore the varying degrees of kidney adverse events observed in the context of CAR T-cell therapy across different trials and indications.

**Table 1:** Renal adverse events in various CAR T-cell trials.

Trial name	Indication	Kidney adverse events
KARMMA 3 (15)	Multiple myeloma	3% (versus 2% on SOC)
KARMMA 1 (10)	Multiple myeloma	No relevant findings (no SAE)
CARTITUDE 1 (11)	Multiple myeloma	No relevant findings (no SAE)
CARTITUDE 4 (8)	Multiple myeloma	1% (versus 1.4% SOC)
JULIET (16)	Non-Hodgkin lymphoma	Increased blood creatinine level (any grade) 11%, SAE 4% (no SAE)
ZUMA 1 (17)	Non-Hodgkin lymphoma	No relevant findings (no SAE)
ZUMA 7 (18)	Non-Hodgkin lymphoma	Acute kidney injury 2% all + 1% SAE versus SOC 5% all + 2% SAE
BELINDA (19)	Non-Hodgkin lymphoma	Creatinine increase 17.9% all + 1.2% SAE versus SOC 18.1% all + 0.6% SAE

SOC, standard of care; SAE, serious adverse events.

According to the WHO safety database, as of July 24, 2022, VigiBase^®^ accumulated 224 reports concerning CAR T cells within the “acute renal failure” Standardized MedDRA Query (SMQ) category, representing 3.3% of the overall 6,832 CAR T-cell reports. Notably, while they are smaller cohort studies, each centered around roughly 15 acute renal failure (ARF) cases have noted ARF in up to 30% of patients; the present data indicate a lower incidence of 3.3%. CAR T-cell therapies reported most frequently were tisa-cel (108 reports, 48.2%, including 36 pediatric cases), axi-cel (82, 36.6%), and brexu-cel (17, 7.6%) (20, 21).

All the reports were classified as serious, encompassing approximately 54% of cases of deaths. Among those who survived, 57.3% were either recovering or had recovered. The heightened fatality rate (>50%) observed in ARF patients associated with CAR T cells is likely linked to systemic complications in high-risk individuals rather than acute kidney injury (AKI) itself. These complications, such as sepsis, may contribute to the development of AKI. In contrast, more than half of the surviving patients restored their baseline kidney function, emphasizing the positive intrinsic renal prognosis of AKI connected to CAR T-cell therapy, consistent with findings from earlier cohorts (14, 21).

## 4 CAR T-cell journey

### 4.1 The role of fludarabine as part of a combination of lymphodepletion therapy prior to CAR T-cell infusion

Preceding the administration of CAR T-cell therapy, lymphodepletion is necessary to reduce tumor burden, thus preventing the swift exhaustion of CAR T cells and depleting the patient’s T cells to establish a favorable milieu for the engraftment of CAR T-cell infusions. Another important objective of lymphodepletion is to prepare and modify the microenvironment and soluble factors to ensure optimal engraftment. The most commonly used lymphodepletion regimen is a combination of fludarabine and cyclophosphamide for 3 days, usually ending 3 to 5 days before the infusion of the CAR T cells. The use of cyclophosphamide and mesna are already well-established protocols. However, due to the potential risk of kidney injury, there is a need to adjust the dose of fludarabine in specific patients, although it is still inadequately established (**Table 2**) (22–24).

**Table 2:** Adjustment in fludarabine dose recommended in adults (22–24).

Cr Clearance (mL/min)	Fludarabine dose (mg/m²)
≥ 80	25 (full dose)
50–79	20
30–49	15
<30	Intravenous formulation, not used Or Use of oral formulation with 50% adjustment Or Change fludarabine to bendamustin

Several crucial points regarding fludarabine as a lymphodepletion therapy merit consideration (22–24):

- Currently, it remains the primary drug in lymphodepletion, and it appears unlikely that a superior alternative will emerge in the foreseeable future.
- The pharmacokinetics and pharmacodynamics of the medication exhibit variations between adults and children.
- Caution is advised when contemplating fludarabine treatment for individuals with pre-existing renal dysfunction, given its predominant elimination through urinary excretion.
- Practical challenges arise as there is currently no established method for testing serum levels of the medication.

There is a lack of unanimity in dose adjustments, although the most widely accepted approaches are outlined here. The AUC (area under the curve) of fludarabine plays a crucial role in CAR T-cell expansion and activity. In pediatric cases, the identified optimal AUC was found to be ≥13.8 mgh/L, while for adults, according to Scordo et al. in their article on patients undergoing standard Flu/Cy (fludarabine/cyclophosphamide) chemotherapy before axi-cel for relapsed/refractory aggressive B-cell non-Hodgkin lymphoma (B-NHL), the target optimal AUC ranged from 18–20 mgh/L. Interestingly, unlike in children, data in adults indicated that a low AUC (<18 mgh/L) correlated with an increased risk of relapse, while a high AUC (>20 mgh/L) was associated with a higher incidence of immune effector cells-associated neurotoxicity syndrome (ICANS) events. Consequently, maintaining the optimal AUC within the range of 18–20 mgh/L demonstrated improved survival outcomes and a safer tolerability profile (9). In essence, this complexity adds a layer of challenge to management, as identifying the precise optimal AUC level remains highly challenging in clinical practice. When fludarabine cannot be utilized, there is no distinct substitute readily available. However, bendamustine has emerged as the most frequently prescribed drug in such scenarios (22–24).

### 4.2 Early post-infusion period

After 2–3 weeks following the infusion of CAR T cells, the patient is at major risk of acute toxicities. Renal toxicity can occur because of drug toxicity (e.g., fludarabine) or as a result of CAR T-cell-related inflammatory syndromes (25).

#### 4.2.1 Cytokine release syndrome

This is the most common severe reaction to CAR T cells. CRS is generated by the secretion of inflammatory cytokines (mainly IL-1, TNF-alpha, and IL-6) by activated CAR T cells and other immune cells involved in the antitumor response (e.g., macrophages and B cells). Over 75% of patients treated with CAR T-cell therapy will develop CRS and approximately 25%–50% will develop a severe CRS, with the greatest risk factor being a high tumor burden. The onset is typically within a few days of the first infusion and takes approximately 1 week to resolve on average, depending on the disease, patient, CAR T-cell type, and CRS severity (3, 25).

According to prior studies, a five- to ten-fold increase in the risk of developing acute kidney injury (AKI) is associated with high-grade CRS. In this context, AKI results from vasodilation driven by inflammatory cytokines, vascular leakage, and subsequent reduction in renal perfusion leading to reduced renal function (14, 26–28).

The fundamentals of CRS management are similar to those required for septic shock, and include broad-spectrum antibiotics (when severe neutropenic), intravenous fluids and antipyretics, and vasopressors, as needed. However, in contrast to septic shock, the cytokines are directly targeted with an interleukin-6 antagonist, either tocilizumab (an IL-6 receptor antibody) or other IL inhibitors such as siltuximab (a direct IL-6 antibody) (28).

#### 4.2.2 Immune effector cells-associated neurotoxicity syndrome

ICANS is a condition that can occur after CAR T-cell therapy and is not directly associated with AKI. It is characterized by a range of neurological symptoms, including confusion, seizures, and speech difficulties (2). The exact mechanisms responsible for severe ICANS after CD19 CAR T-cell therapy are not well understood (3). Treatment for ICANS typically involves supportive care, administration of corticosteroids, and a dedicated neurology team (1, 25).

#### 4.2.3 Immune effector cells-associated hemophagocytic lymphohistiocytosis-like syndrome

IEC-associated hemophagocytic lymphohistiocytosis-like syndrome (IEC-HS) can occur after CAR T-cell therapy. Hemophagocytic lymphohistiocytosis (HLH) is a rare but serious event associated with CAR T-cell treatment. It is characterized by severe immune activation and immune-related organ failure. In individuals with hemophagocytic lymphohistiocytosis, there could be a manifestation of collapsing glomerulopathy (29), and in this scenario, it may have occurred due to cytokine-triggered damage to podocytes and endothelial cells (30, 31). Diagnosis can be difficult, and the best course of treatment is not always clear. The most common symptoms are pancytopenia, liver and kidney dysfunction, hepatosplenomegaly, and fever.

The spectrum of this disease includes tumor lysis syndrome (TLS), which is characterized by high blood potassium, high blood phosphate, low blood calcium, high blood uric acid, and higher than normal levels of blood urea nitrogen. These changes in blood electrolytes and metabolites are a result of the release of cellular contents of dying cells into the bloodstream. TLS can ultimately result in serious complications such as acute uric acid nephropathy, acute kidney failure, seizures, and cardiac arrhythmias (25). The main identified causes of tumor lysis syndrome (TLS)-induced acute kidney injury (AKI) involve crystal nephropathies arising from the deposition of uric acid, xanthine, and/or calcium phosphate crystals within renal tubules. However, in this situation, alternative mechanisms can also contribute to acute tubular necrosis. These include the liberation of pro-inflammatory cytokines affecting renal microcirculation, as well as tubulointerstitial damage caused by tumor infiltration into the renal parenchyma or compression of the ureter by the tumor mass (32).

### 4.3 Late follow-up

After 3–4 weeks post-CAR T-cell infusion, the patient initiates outpatient visits, occurring approximately every 1–2 weeks. During this phase, the primary emphasis is placed on the long-term monitoring of cytopenias, assessment of immunodeficiency with a focus on CD19 and IgG levels, and vigilance against related infections. Disease monitoring also becomes a central aspect of care during these follow-up visits. It is noteworthy that after 8–12 weeks, acute kidney injury (AKI) becomes a rare manifestation, given that the majority of patients experience recovery, with kidney function returning to baseline within 30 days. This underscores the dynamic nature of potential adverse events throughout the course of CAR T-cell therapy (1, 14, 25).

## 5 Discussion

In the realm of CAR T-cell therapy, nephrologists familiar with bone marrow transplantation recognize some commonalities. Apheresis in CAR T-cell therapy resembles that in transplants, and the conditioning phase, often based on fludarabine, shares similarities.

However, significant differences emerge post-infusion in CAR T-cell therapy. Unlike post-transplant scenarios, there is no graft-versus-host disease (GVHD) following CAR T-cell treatment; neutropenia is typically shorter, mucositis is absent, and the level of infections is lower compared to allogeneic transplants. Nonetheless, CAR T-cell therapy introduces a range of new complications and syndromes, unique to this treatment and novel to healthcare professionals, given the therapy’s recent development.

The role of the nephrologist in managing patients undergoing CAR T-cell therapy is twofold and crucial. Nephrologists can assist in managing renal toxicities when they arise, which, as discussed, is not always the case. More importantly, in our view, is their role in aiding with the differential diagnosis of these toxicities. Conditions like cytokine release syndrome (CRS), hemophagocytic syndrome, ICANS, sepsis, and drug toxicity can all lead to acute kidney injury, presenting diagnostic challenges for hematologists and affecting patient management.

The complexity increases when additional factors come into play. For instance, kidney involvement in multiple myeloma is quite common. Thus, the kidneys may be affected by the underlying disease either prior to or during any phase of the treatment. Post-CAR T-cell therapy, patients often experience hypogammaglobulinemia due to the massive destruction of B cells, which are crucial for defense, particularly in cases of lymphomas, leukemias, and multiple myelomas. These patients frequently require indefinite prophylactic replacement of immunoglobulins. However, to complicate matters, multiple myeloma can also cause a falsely normal or elevated gammaglobulin level, masking an underlying functional hypogammaglobulinemia (33).

## 6 Conclusions

Kidney injury is a significant risk following CAR T-cell infusion, often resulting from drug-related and inflammatory toxicities. While these complications are generally reversible, the expertise of nephrologists is invaluable in mitigating renal damage and effectively managing both common and rare cases of acute kidney injury (AKI). This necessitates a collaborative approach, especially between nephrologists and oncohematologists, to ensure accurate diagnosis and tailored treatment strategies. Such interdisciplinary teamwork not only enhances patient safety but also underscores the vital role of comprehensive care in the evolving landscape of cancer therapy. Through these concerted efforts, we can improve treatment outcomes and navigate the complexities associated with CAR T-cell therapy.

## Author contributions

MS: Conceptualization, Data curation, Formal analysis, Investigation, Methodology, Project administration, Resources, Software, Supervision, Validation, Visualization, Writing – original draft, Writing – review & editing. AM: Formal analysis, Supervision, Validation, Writing – review & editing. DR: Data curation, Methodology, Project administration, Supervision, Validation, Visualization, Writing – original draft, Writing – review & editing, Conceptualization, Formal analysis, Investigation, Resources, Software. MP: Data curation, Supervision, Validation, Visualization, Writing – review & editing. AP: Validation, Visualization, Writing – review & editing. AC: Validation, Visualization, Writing – review & editing. AS: Data curation, Methodology, Project administration, Supervision, Validation, Visualization, Writing – original draft, Writing – review & editing.
